# A Case of Acute Abscess of the Liver, Opened through the Abdominal Walls; with Recovery

**Published:** 1881-03

**Authors:** Samuel J. Holmes

**Affiliations:** 313 Fulton St., Chicago


### Article VI.

### A Case of Acute Abscess of the Liver, Opened Through the Abdominal Walls; with Recovery. By Samuel J. Holmes, M.D.

I regret that I am obliged to give the history of the case, substantially as I recall it from memory, not having kept a diurnal history, but if I was able to present it more in detail, perhaps it would furnish no *special* features, as differing from the usual case of acute abscess of the liver.

It is interesting as an extremely rare affection, in the temperate climate, and one belonging, *par excellence*, to the warm, and is still further interesting, as illustrating the recovery of a patient, even under the adverse circumstances of age, and the breaking down of a large tract of glandular structure, in an organ so important as the liver, and seems to indicate the advisability of early operative procedure (to evacuate the abscess cavity), as soon as the liver can be clearly defined, as having become adherent to the abdominal walls. The cessation of the patient's discomfort, and the gradual but constant recovery from the weakened condition into which she had necessarily sunk, prove the operation to be a measure of economy, in the preservation of strength, for to have put off the opening of the abscess until nature should have created an opening, would have been a positive prolonging of the patient's suffering, insured in a measure her exhaustion (as her physical forces were fast succumbing) and would eventually have proven fatal.

History: Mrs. B., of 53 N. May street, aged fifty-nine years, of full habit, called me in the latter part of August. The patient suffered an attack of malarial fever, about thirty years ago, since which time she has enjoyed comparatively good health. Eight years ago she became cognizant of an enlargement within the abdomen, which she supposed to be a tumor, but felt no further inconvenience or discomfort from the same, other than a sensation of pulsation. She could not say that she had been aware of its increase in size since that time. Two weeks previous to seeing me, she had visited friends in Wisconsin, where malaria was prevailing, and feeling unsafe here, returned to her home in Chicago. Three days previous to summoning a physician, she crossed the lake to South Haven, returning the following day, and suffered extreme sea-sickness both in crossing and returning. After returning, she ate injudiciously of fruit, and upon the following day I saw her with these symptoms presented: Vomiting; pain, quite severe in character, referred to the epigastric region; tenderness over the same region; constipation of the bowels; coated tongue, fever, and an acceleration of the pulse. My attention was directed to the enlargement within the abdomen, which by palpation and percussion I was enabled to define as follows: Easily traceable to three inches below the umbilicus—and beneath the ribs above the umbilicus—to the left of the median line above and below the umbilicus (with a zone of percussion resonance between the enlargement and spleen) and to the right of the median line above the umbilicus, the surface was smooth, and its inferior extremity blunt and rounded. It will be seen that the enlargement extended below the umbilicus, while the tenderness, as before indicated, was limited to that part above the umbilicus corresponding to the epigastric region. I concluded that the enlargement was that of the left lobe of the liver, and by differentiation, it was fair to suppose, one of hypertrophy, from malarial causes of years previous. Fatty liver, amyloid liver, and carcinoma, were readily excluded, and as no fluctuation was discernable, the idea of a cyst, of hydated nature, or chronic abscess, was not to be entertained.

Enlargement of the liver frequently, but not invariably, exists in abscess of the liver, and the enlargement may be found to be limited to a particular direction, and not affecting the whole organ. But the acute symptoms were referred to the stomach, considering the immediate exciting causes which would be calculated to disturb that organ with such symptoms as were manifest in the case, and so the liver was excused from hepatitis, of a suppurative nature, and the nearest approach, or conjecture (for it could be but conjectural at this time) was the thought, that perhaps there might exist a perihepatitis, or circumscribed peritoneal inflammation of that portion of the liver from which the pain and tenderness arose.

The patient was given a saline cathartic (which did not operate), bismuth and pepsin to control vomiting, quinine to allay the fever, an opiate to control pain, and a sinapism was applied over the epigastric region, followed by hot flaxseed meal poultices continuously applied. These remedies were administered in behalf of the stomach, but if hepatitis had been clearly made out, would not have been inappropriate as treatment. The symptoms continued for about one week, with varying intensity, and no change in the administration of medicine, except to change the cathartic, and finding that the greatest obstinacy of the bowels obtained, to effect a movement later by enemas. The amount of morphine required to control the pain was large, which first created the suspicion that the pain was too intense for a catarrhal condition of the stomach. By request of the family, Prof. Byford was called in consultation, and, after a careful examination, rendered the diagnosis of hypertrophy of the liver, and referred the acute symptoms to the stomach, a diagnosis in accord with the first made. His only suggestion was to supplant the morphine by hydrate of chloral, and the administration of a small amount of calomel, which was carried out. Another week passed, and the symptoms changed materially. The fever which had been continuous, became intermitting, with frequent rigors through the day, and sweating (all of which symptoms were irrespective of the time of day) and the patient growing weaker. It seemed apparent now, that pus was confined in some part of the body, and, to a probability, the lower. Very soon, a slight pouting, or prominence manifested itself about three inches above the umbilicus, corresponding to a point near the median line, which soon took on redness, and over which a sense of fluctuation was discernable. This became larger and larger, and gave every evidence of an abscess of the liver. Prof. Byford again saw the case with me, and did not question the presence of an hepatic abscess. The patient was weak and debilitated, and a few days previous, I placed her upon calisaya bark and wine, continuing the quinine, and dispensing with all other medicine except the chloral. Satisfied that the anterior surface of the liver was adherent to the peritoneal surface of the abdominal walls (and the patient growing constantly weaker), I advised her to allow of its being opened, but she preferred to await the powers of nature to consummate the same, and so deferred. A few days passed, and becoming apparently exhausted, and disappointed in the expedition with which nature ulcerated its way through the walls of the abdomen (the anterior wall of the abscess), she reluctantly submitted to an operative procedure, which was to extenuate her suffering, and place her in a condition to recover. I opened the abscess freely, selecting a point as near the center of the prominence, over which fluctuation was to be felt, as possible (for there was no point which manifested any degree of thinning of the wall over that of other parts of the same), and drained from the cavity from six to to eight ounces of pus, introduced a drainage tube, and covered the whole with oakum. The abscess was dressed twice a day, and the wine and bark given freely. The effect of opening the abscess was a most salutary one. Rigors, fever and sweating, ceased immediately. Sleep, natural and refreshing, came to assist in the return of strength, appetite fast improved, and the change in the patient's general condition was such as to win from her the greatest commendation in surgical interference, for she termed it "her new lease of life." The abscess cavity, after the first week, slowly but progressively filled in from the bottom; the drainage tube was frequently shortened; and in five weeks from the time of opening the abscess, the cavity was closed; the patient walking about (in spite of the excessive drain), but with some remaining asthenia.

Microscopic examination of the pus, revealed in addition to ordinary constituents of pus, cholesterine crystals, and disintegrated hepatic tissue.

The diagnosis of the affection is obscured early in its history, by the infrequency of the disease, in our climate, and the equivocal symptoms which accompany it. Indeed, it cannot be made with any degree of certainty, previous to the discharge of pus into one of the cavities of the body (with its subsequent elimination externally to the sight), or its manifestation upon the abdominal walls, where manual exploration reveals the true nature of the disease. It is true, that the clinical symptoms of the disease, in the warm climate, would doubtless arouse the suspicion of the disease being present, but as I have before said, with us it would be equally rare, to venture even the supposition, before the time when it asserts itself, and makes unnecessary any hypothesis, and again the symptoms which precede the fluctuating enlargement, are not always well marked.

Nature is indeed conservative in advancing the abscess to this issue, and as we think of the situations in which it *might* have ruptured, with a positive fatal termination, it lends an opportunity to applaud her efforts. The bronchial tubes and alimentary canal are next in order as to favorable localities for evacuating the pus, and it might be evacuated into the right pleural cavity, and subsequently into the lung of the same side, with favorable termination; but the peritoneal cavity, the pericardial cavity, or hepatic vein, and inferior vena cava, are receptacles which furnish, as a rule, fatal results. We can scarcely expect a favorable issue, even when the abscess is evacuated in the *most favorable* direction, and multiple abscesses render the prognosis most grave, as is apparent.

The case made a quick recovery, if statistics as to the average duration are reliable. Of course the direction taken by the pus, determines the time required for recovery. The average duration would seem to be from four to five months, and the longest time is required where the discharge is through the integument. From observation of cases, it would seem that the largest number of cases occur in the right lobe (the left in this), and most frequently in the posterior portion (anterior in the case considered).

To arrive at the special factors of etiology, is a matter of great difficulty. I do not think that it was possible for the patient to have sustained a contusion, sufficient to have caused the abscess, in crossing the lake, although she describes the "shaking and pounding" as of considerable moment. I do not remember to have read or heard of a case in which a chronic congestion of the liver, became kindled into an activity sufficient to have eventuated in abscess, but the liver in this case, was hypertrophied, and doubtless in a state of chronic congestion, and I am not disinclined to look upon this feature as enacting some part, or sustaining some causative relation, in the etiology of this case. I do not think that a visit to a malarial country had to do with reinforcing a previous existing condition, for the patient suffered no malarial fever subsequently. Sufficient uniformity in cases as to their apparent etiology is a desideratum, and the case ranks admirably with precedent in this regard.

313 Fulton St., Chicago.

